# Association of Genetic Variants in Complement Factor H and Factor H-Related Genes with Systemic Lupus Erythematosus Susceptibility

**DOI:** 10.1371/journal.pgen.1002079

**Published:** 2011-05-26

**Authors:** Jian Zhao, Hui Wu, Melanie Khosravi, Huijuan Cui, Xiaoxia Qian, Jennifer A. Kelly, Kenneth M. Kaufman, Carl D. Langefeld, Adrienne H. Williams, Mary E. Comeau, Julie T. Ziegler, Miranda C. Marion, Adam Adler, Stuart B. Glenn, Marta E. Alarcón-Riquelme, BIOLUPUS Network, GENLES Network, Bernardo A. Pons-Estel, John B. Harley, Sang-Cheol Bae, So-Young Bang, Soo-Kyung Cho, Chaim O. Jacob, Timothy J. Vyse, Timothy B. Niewold, Patrick M. Gaffney, Kathy L. Moser, Robert P. Kimberly, Jeffrey C. Edberg, Elizabeth E. Brown, Graciela S. Alarcon, Michelle A. Petri, Rosalind Ramsey-Goldman, Luis M. Vilá, John D. Reveille, Judith A. James, Gary S. Gilkeson, Diane L. Kamen, Barry I. Freedman, Juan-Manuel Anaya, Joan T. Merrill, Lindsey A. Criswell, R. Hal Scofield, Anne M. Stevens, Joel M. Guthridge, Deh-Ming Chang, Yeong Wook Song, Ji Ah Park, Eun Young Lee, Susan A. Boackle, Jennifer M. Grossman, Bevra H. Hahn, Timothy H. J. Goodship, Rita M. Cantor, Chack-Yung Yu, Nan Shen, Betty P. Tsao

**Affiliations:** 1 Division of Rheumatology, Department of Medicine, David Geffen School of Medicine, University of California Los Angeles, Los Angeles, California, United States of America; 2 Joint Molecular Rheumatology Laboratory of Institute of Health Sciences and Shanghai Renji Hospital, Shanghai Jiao Tong University School of Medicine, Shanghai Institutes for Biological Sciences, and Chinese Academy of Sciences, Shanghai, China; 3 Arthritis and Clinical Immunology Research Program, Oklahoma Medical Research Foundation, Oklahoma City, Oklahoma, United States of America; 4 United States Department of Veterans Affairs Medical Center, Oklahoma City, Oklahoma, United States of America; 5 Department of Biostatistical Sciences, Wake Forest University Health Sciences, Wake Forest, North Carolina, United States of America; 6 Center for Genomics and Oncological Research, Pfizer-University of Granada-Junta de Andalucia, Granada, Spain; 7 Sanatorio Parque, Rosario, Argentina; 8 Cincinnati Children's Hospital Medical Center, Cincinnati, Ohio, United States of America; 9 United States Department of Veterans Affairs Medical Center, Cincinnati, Ohio, United States of America; 10 Department of Rheumatology, Hanyang University Hospital for Rheumatic Diseases, Seoul, Korea; 11 Department of Medicine, Keck School of Medicine, University of Southern California, Los Angeles, California, United States of America; 12 Divisions of Genetics and Molecular Medicine and Immunology, King's College London, London, United Kingdom; 13 Section of Rheumatology and Gwen Knapp Center for Lupus and Immunology Research, University of Chicago, Chicago, Illinois, United States of America; 14 Department of Medicine, University of Alabama at Birmingham, Birmingham, Alabama, United States of America; 15 Department of Epidemiology, University of Alabama at Birmingham, Birmingham, Alabama, United States of America; 16 Department of Medicine, Johns Hopkins University School of Medicine, Baltimore, Maryland, United States of America; 17 Division of Rheumatology, Feinberg School of Medicine, Northwestern University, Chicago, Illinois, United States of America; 18 Division of Rheumatology, Department of Medicine, University of Puerto Rico Medical Sciences Campus, San Juan, Puerto Rico; 19 Rheumatology and Clinical Immunogenetics, University of Texas Health Science Center at Houston, Houston, Texas, United States of America; 20 Department of Medicine, University of Oklahoma Health Sciences Center, Oklahoma City, Oklahoma, United States of America; 21 Division of Rheumatology, Medical University of South Carolina, Charleston, South Carolina, United States of America; 22 Department of Internal Medicine, Wake Forest University Health Sciences, Winston-Salem, North Carolina, United States of America; 23 Center for Autoimmune Disease Research, Universidad del Rosario, Bogota, Colombia; 24 Clinical Pharmacology, Oklahoma Medical Research Foundation, Oklahoma City, Oklahoma, United States of America; 25 Rosalind Russell Medical Research Center for Arthritis, Department of Medicine, University of California San Francisco, San Francisco, California, United States of America; 26 Division of Rheumatology, Department of Pediatrics, University of Washington, Seattle, Washington, United States of America; 27 Center for Immunity and Immunotherapies, Seattle Children's Research Institute, Seattle, Washington, United States of America; 28 National Defense Medical Center, Taipei, Taiwan; 29 Division of Rheumatology, Seoul National University, Seoul, Korea; 30 Division of Rheumatology, School of Medicine, University of Colorado Denver, Aurora, Colorado, United States of America; 31 Institute of Human Genetics, Newcastle University, Newcastle upon Tyne, United Kingdom; 32 Department of Human Genetics, University of California Los Angeles, Los Angeles, California, United States of America; 33 Department of Pediatrics, The Ohio State University, Columbus, Ohio, United States of America; University of Liège, Belgium

## Abstract

Systemic lupus erythematosus (SLE), a complex polygenic autoimmune disease, is associated with increased complement activation. Variants of genes encoding complement regulator factor H (CFH) and five CFH-related proteins (CFHR1-CFHR5) within the chromosome 1q32 locus linked to SLE, have been associated with multiple human diseases and may contribute to dysregulated complement activation predisposing to SLE. We assessed 60 SNPs covering the *CFH*-*CFHRs* region for association with SLE in 15,864 case-control subjects derived from four ethnic groups. Significant allelic associations with SLE were detected in European Americans (EA) and African Americans (AA), which could be attributed to an intronic *CFH* SNP (rs6677604, in intron 11, *P*_meta_ = 6.6×10^−8^, OR = 1.18) and an intergenic SNP between *CFHR1* and *CFHR4* (rs16840639, *P*_meta_ = 2.9×10^−7^, OR = 1.17) rather than to previously identified disease-associated *CFH* exonic SNPs, including I62V, Y402H, A474A, and D936E. In addition, allelic association of rs6677604 with SLE was subsequently confirmed in Asians (AS). Haplotype analysis revealed that the underlying causal variant, tagged by rs6677604 and rs16840639, was localized to a ∼146 kb block extending from intron 9 of *CFH* to downstream of *CFHR1*. Within this block, the deletion of *CFHR3* and *CFHR1* (*CFHR3-1*Δ), a likely causal variant measured using multiplex ligation-dependent probe amplification, was tagged by rs6677604 in EA and AS and rs16840639 in AA, respectively. Deduced from genotypic associations of tag SNPs in EA, AA, and AS, homozygous deletion of *CFHR3-1*Δ (*P*_meta_ = 3.2×10^−7^, OR = 1.47) conferred a higher risk of SLE than heterozygous deletion (*P*_meta_ = 3.5×10^−4^, OR = 1.14). These results suggested that the *CFHR3-1*Δ deletion within the SLE-associated block, but not the previously described exonic SNPs of *CFH*, might contribute to the development of SLE in EA, AA, and AS, providing new insights into the role of complement regulators in the pathogenesis of SLE.

## Author Summary

Systemic lupus erythematosus (SLE) is a complex autoimmune disease, associated with increased complement activation. Previous studies have provided evidence for the presence of SLE susceptibility gene(s) in the chromosome 1q31-32 locus. Within 1q32, genes encoding complement regulator factor H (CFH) and five CFH-related proteins (CFHR1-CFHR5) may contribute to the development of SLE, because genetic variants of these genes impair complement regulation and predispose to various human diseases. In this study, we tested association of genetic variants in the region containing *CFH* and *CFHRs* with SLE. We identified genetic variants predisposing to SLE in European American, African American, and Asian populations, which might be attributed to the deletion of *CFHR3* and *CFHR1* genes but not previously identified disease-associated exonic variants of *CFH*. This study provides the first evidence for consistent association between *CFH/CFHRs* and SLE across multi-ancestral SLE datasets, providing new insights into the role of complement regulators in the pathogenesis of SLE.

## Introduction

SLE (OMIM 152700) is a debilitating autoimmune disease with strong genetic and environmental components, characterized by the production of autoantibodies resulting in tissue injury of multiple organs [1]. In SLE patients, aberrant complement activation leads to inflammatory injury [2], and fluctuation of serum C3 is a commonly used clinical biomarker of SLE disease activity [3]. In addition, a hereditary deficiency of *C1q*, *C1r*, *C1s*, *C4* or *C2* of the classical complement pathway impairs the clearance of immune complexes and debris from apoptotic cells, which strongly predisposes to SLE susceptibility [2]. Common variants of *C3* and *C4* have also been associated with risk of SLE [4], [5], [6]. Collectively, these findings indicate the important role of complement in the development of SLE.

Complement factor H (CFH), a key regulator of the alternative complement pathway, modulates the innate immune responses to microorganisms, controls C3 activation and prevents inflammatory injury to self tissue [7], [8]. CFH inhibits complement activation by preventing the formation and accelerating the decay of C3 convertase and acting as a cofactor for factor I-mediated degradation of C3b, both in plasma and on cell surfaces. Structurally, CFH contains 20 short consensus repeats (SCRs). SCR1-4 in the N-terminus mediate the cofactor/decay accelerating activity and SCR19-20 in the C-terminus are essential for cell surface regulation of CFH. In addition, CFH contains specific binding sites for polyanion (heparin or sialic acid), C-reactive protein (CRP) and microorganisms. CFH has five related proteins (CFHR1-5), all of which are also composed of SCRs [9]. SCRs in the N-terminus and C-terminus of CFHRs are highly homologous to SCR6-9 and SCR19-20 of CFH, respectively, suggesting that CFHRs and CFH may compete for binding to ligands. CFHRs lack SCRs homologous to SCR1-4 of CFH, and consequently do not exhibit cofactor/decay accelerating activity. Distinct from CFH, CFHR1 can inhibit C5 convertase activity and the formation of terminal membrane attack complex (MAC) [10]. A recent study has shown that CFH deficiency accelerates the development of lupus nephritis in lupus-prone mice MRL-lpr [11]. However, the role of CFHRs in the pathogenesis of SLE is still unknown.

*CFH*, *CFHR3*, *CFHR1*, *CFHR4*, *CFHR2 and CFHR5*, that present in tandem as a gene cluster located in human chromosome 1q32, are positional candidate genes within the 1q31-32 genomic region linked to SLE [12], [13]. In recent years, multiple exonic SNPs in *CFH*, such as I62V, Y402H, D936E and A473A, have been specifically associated with various human diseases including age-related macular degeneration (AMD) [14], [15], atypical hemolytic uremic syndrome (aHUS) [16] and membranoproliferative glomerulonephritis type II (MPGN II) [16], [17] as well as host susceptibility to meningococcal disease [18]. In addition, a common deletion of *CFHR3* and *CFHR1* (*CFHR3-1*Δ) has been associated with increased risk of aHUS [19] and decreased risk of AMD [20]. Taken together, these data prompted us to test whether genetic variants in *CFH* and *CFHRs* predisposed to SLE susceptibility.

Although recent genome wide association studies (GWAS) have b`n successfully used to identify SLE susceptibility genes [21], they still may be underpowered for specific genomic regions due to many factors such as sample size, marker density, ethnicity of subjects and over-stringent significance threshold. In these cases, a well-designed candidate gene-based association study can be used as a complementary approach to GWAS to identify genetic variants with modest effect size.

In this study, we fine mapped the *CFH*-*CFHRs* region using 60 SNPs and assessed their association with SLE susceptibility in a collection of 15,864 subjects (8,372 cases vs. 7,492 controls) from four ethnic groups. In addition, we assessed the association of *CFHR3-1*Δ with SLE by using tag SNPs.

## Results

### SNPs in the *CFH-CFHRs* region were associated with SLE susceptibility in European Americans and African Americans

**Figure 1:**
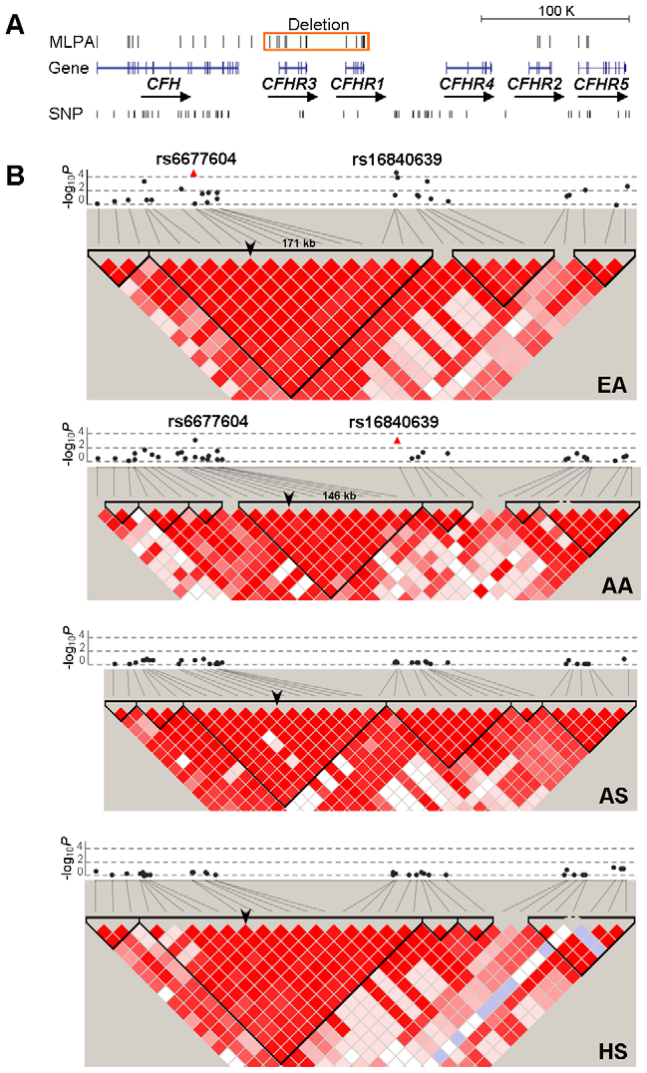
Allelic association of SNPs in the *CFH-CFHRs* region with SLE and their LD patterns. A) The genomic structure of the *CFH-CFHRs* region and the location of all SNP and MLPA markers are indicated. The deletion of *CFHR3* and *CFHR1* detected by MLPA markers is shown as a red box. B) The allelic *P* value of each SNP with SLE (−log_10_ *P*) is plotted as a black circle according to its coordinate. The two SNPs exhibiting the strongest association with SLE in EA (rs6677604) and AA (rs16846039) are highlighted as red triangles. SNPs that failed in the HWE testing or showed low genotyping quality are not shown. SNP-constructed haplotype blocks were defined by Haploview using the confidence intervals model. An arrowhead is used to indicate the position of rs6677604 in the haplotype blocks.

To assess the association of *CFH* and *CFHRs* genes with SLE, we genotyped 60 tag SNPs covering the ∼360 kb *CFH*-*CFHRs* region in unrelated case-control subjects derived from four ethnic groups including European Americans (EA), African Americans (AA), Asians (AS), and Hispanics enriched for the Amerindian-European admixture (HS) (Figure 1A) (Table S1). According to the latest Hapmap CEU dataset (release 28), within the *CFH*-*CFHRs* region, 203 of 224 (90%) common SNPs (frequency>5%) could be captured by SNPs used in this study with r^2^>0.70. Within the most-studied gene *CFH*, previously identified disease-associated exonic SNPs including I62V (rs800292, typed), Y402H (tagged by rs7529589), D936E (tagged by rs10489456) and A474A (tagged by rs1410996) were evaluated for the association with SLE.

In the largest dataset (3,936 EA cases vs. 3,491 EA controls), after removing those failing the Hardy-Weinberg equilibrium (HWE) testing or showing low genotyping quality, fourteen SNPs were significantly associated with SLE (allelic *P*<0.05) (Table 1), of which rs6677604, located in intron 11 of *CFH*, exhibited the strongest association signal (minor allele frequency [MAF]: 23.0% in case vs. 20.1% in control, *P* = 2.4×10^−5^, OR[95%CI] = 1.19[1.10–1.28]). In the second largest dataset (1,679 AA cases vs. 1,934 AA controls), four SNPs were significantly associated with SLE (Table 1), all of which confirmed the association detected in EA, with rs16840639, located in the intergenic region between *CFHR1* and *CFHR4*, showing the strongest association signal with a similar effect size (MAF: 37.5% vs. 33.7%, *P* = 6.6×10^−4^, OR[95%CI] = 1.18[1.07–1.31]). After Bonferroni correction for multiple comparisons, the association of rs6677604 and rs16840639 with SLE remained significant in both EA and AA (Table 1). However, in the two smaller datasets (1,265 AS cases vs. 1,260 AS controls and 1,492 HS cases vs. 807 HS controls), we failed to detect significant association of these SNPs with SLE (Table S1).

**Table 1:** Association of SNPs in the *CFH-CFHRs* region with SLE in European Americans and African Americans.

	Coordinate on Chr1		Tested	Freq				Conditional analysis *P*	
SNP	and location on gene	Ethnicity	allele	SLE	Control	OR [95% CI]	Allelic *P*	rs6677604	rs16840639
rs16840422	194919457	EA	A	16.5%	14.4%	1.17[1.07–1.28]	***4.8*** **×** ***10^−4^***	0.89	1.00
	*CFH* intron 6	AA	A	44.0%	41.3%	1.12[1.02–1.23]	0.020	0.29	0.79
rs203685	194944568	EA	C	39.5%	41.9%	0.91[0.85–0.97]	4.0×10^−3^	0.32	0.33
	*CFH* intron 9	AA	C	20.4%	22.3%	0.90[0.80–1.00]	0.058	-	-
rs6677604	194953541	EA	A	23.0%	20.1%	1.19[1.10–1.28]	***2.4*** **×** ***10^−5^***	-	NV
	*CFH* intron 11	AA	A	38.8%	35.0%	1.18[1.07–1.30]	***7.5*** **×** ***10^−4^***	-	0.18
rs381974	194959295	EA	A	39.4%	41.2%	0.93[0.87–0.99]	0.025	0.61	0.59
	*CFH* intron 11	AA	A	25.0%	26.0%	0.95[0.85–1.05]	0.32	-	-
rs1410996	194963556	EA	A	42.9%	40.9%	1.09[1.02–1.16]	0.015	0.80	0.79
	*CFH* intron 14	AA	A	56.4%	54.8%	1.07[0.97–1.17]	0.18	-	-
rs1329428	194969433	EA	A	42.6%	40.7%	1.08[1.02–1.16]	0.017	0.74	0.73
	*CFH* intron 15	AA	A	49.1%	46.6%	1.10[1.01–1.21]	0.036	0.65	0.98
rs10922144	195089923	EA	A	19.4%	20.7%	0.92[0.85–1.00]	0.047	0.32	0.35
	*CFHR4* 5′ upstream	AA	A	5.7%	6.2%	0.92[0.76–1.12]	0.41*	-	-
rs7542235	195090236	EA	G	23.1%	20.2%	1.18[1.09–1.28]	***2.6*** **×** ***10^−5^***	NV	NV
	*CFHR4* 5′ upstream	AA	G	38.0%	34.4%	1.17[1.06–1.29]	0.0014*	-	-
rs16840639	195091396	EA	G	22.7%	20.1%	1.17[1.08–1.27]	***1.2*** **×** ***10^−4^***	NV	-
	*CFHR4* 5′ upstream	AA	G	37.5%	33.7%	1.18[1.07–1.31]	***6.6*** **×** ***10^−4^***	0.78	-
rs6657442	195104683	EA	G	18.8%	20.1%	0.92[0.85–1.00]	0.043	0.36	0.40
	*CFHR4* 5′ upstream	AA	G	5.1%	5.4%	0.94[0.76–1.16]	0.56*	-	-
rs6428370	195111216	EA	G	36.1%	33.3%	1.13[1.06–1.21]	***3.6*** **×** ***10^−4^***	0.28	0.31
	*CFHR4* 5′ upstream	AA	G	62.1%	59.7%	1.11[1.00–1.22]	0.041*	-	-
rs6667243	195208116	EA	A	47.7%	49.5%	0.93[0.87–0.99]	0.034	0.21	0.28
	*CFHR5* 5′ upstream	AA	A	17.3%	18.2%	0.94[0.84–1.07]	0.35	-	-
rs1759016	195219121	EA	A	35.7%	33.5%	1.10[1.03–1.18]	6.7×10^−3^	0.53	0.58
	*CFHR5* intron 2	AA	A	70.5%	69.2%	1.06[0.96–1.17]	0.25	-	-
rs6663083	195247283	EA	G	47.7%	50.2%	0.90[0.84–0.96]	2.3×10^−3^	0.19	0.19
	*CFHR5* 3′ downstream	AA	G	17.2%	18.4%	0.92[0.82–1.04]	0.20	-	-

Abbreviation: Freq, allele frequency; OR, odds ratio; CI, confidence interval; NV represents that comparison between these two SNPs was not valid in conditional analysis due to their strong LD. Coordinate of each SNP is based on NCBI build 36. SNPs that showed association with SLE (*P*<0.05 before correction) in either EA or AA are listed in this table. If an allelic *P* value remained significant after Bonferroni correction, it is highlighted as bold and italic. For SNPs that failed in HWE test, the allelic *P* value is marked by “*”. For SNPs that were not used in conditional analysis, the conditional *P* value is denoted as “-”. SNPs that did not show association with SLE are listed in Table S1. The HWE *P* value and genotyping missing rate of each SNP are listed in Table S1.

Of note, we did not detect significant association of I62V, Y402H and D936E with SLE in any of the four datasets (Table S1). A474A was associated with risk of SLE in EA (*P* = 0.015 before correction, OR[95%CI] = 1.09[1.02–1.16]), but it was not confirmed in the other three ethnic groups (Table S1).

### The causal variant could be localized to a ∼146 kb block and was tagged by the minor allele of rs6677604 and rs16840639

**Figure 2:**
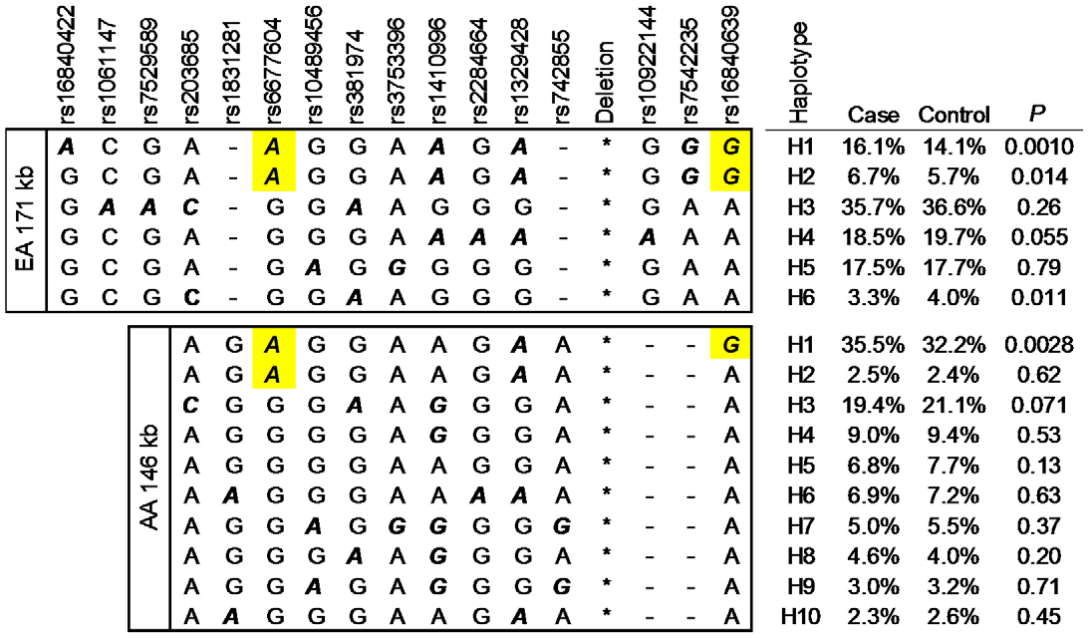
The minor allele of rs6677604 and rs16846039 tag risk haplotypes of SLE. Haplotypes containing rs6677604 and rs16846039 (frequency>1%) are constructed in both EA and AA subjects, which correspond to the 2^nd^ and the 4^th^ block of EA and AA shown in Figure 1, respectively. SNPs not used to construct haplotypes are marked as “-”. The minor alleles of rs6677604 and rs16840639 are highlighted in a yellow box. The minor allele of each SNP is bolded and italicized. The position of *CFHR3-1*Δ is indicated as “*”.

To localize the underlying causal variant, we compared all SLE-associated SNPs (*P*<0.05) identified in EA and AA and carried out linkage equilibrium (LD) analysis. Fourteen SNPs, spanning from intron 6 of *CFH* to the 3′ region downstream of *CFHR5*, were associated with SLE in EA. However, only 4 of 14 SNPs, spanning from intron 6 of *CFH* to the 5′ region upstream of *CFHR4*, showed consistent association with SLE in AA, suggesting a smaller SLE risk region. Of interest, within the risk region, rs6677604 and rs16840639 exhibited the strongest association with SLE in EA and AA, respectively. We found that rs6677604 and rs16840639 were in strong LD with each other in both EA (r^2^ = 0.96) and AA (r^2^ = 0.77). Haplotype analysis showed that rs6677604 and rs16840639 could be defined into a ∼171 kb block in EA and a smaller ∼146 kb block in AA, respectively (Figure 1B). The minor allele of rs6677604 or rs16840639 perfectly tagged two SLE risk haplotypes in EA (H1: 16.1% vs. 14.1%, *P* = 0.0010; H2: 6.7% vs. 5.7%, *P* = 0.014), and the minor allele of rs16840639 perfectly tagged the only risk haplotype in AA (H1: 35.5% vs. 32.2%, *P* = 0.0028) (Figure 2).

Using the conditional haplotype-based association test, we showed that after conditioning on rs6677604 or rs16840639 significant associations of all other SNPs were eliminated in both EA and AA (Table 1), which suggested that rs6677604 and rs16840639 could account for all association signals in the *CFH-CFHRs* region. Due to the strong LD between rs6677604 and rs16840639, the conditional test could not be applied to further distinguish their association signals.

To compare between rs6677604 and rs16840639, we combined their ORs detected in EA and AA to generate a meta-analysis *P* value. The combined *P* value of rs6677604 (*P*_meta_ = 6.6×10^−8^, OR[95%CI] = 1.18[1.11–1.26]) was stronger than that of rs16840639 (*P*_meta_ = 2.9×10^−7^, OR[95%CI] = 1.17[1.10–1.2]).

Taken together, these data suggested that the underlying causal variant of SLE was captured by two strongly SLE-associated SNPs rs6677604 and rs16840639 in this study, which might reside in a ∼146 kb block. Neither rs6677604 nor rs16840639 are located in genomic regions with known biological function, which prompted us to seek other likely causal variants within the SLE-associated block.

### The *CFHR3-1*Δ deletion was tagged by the minor allele of rs6677604 and rs16840639

*CFHR3-1*Δ is a likely functional variant within the ∼146 kb SLE-associated block (as shown in Figure 1A and 1B), which results in the deletion of *CFHR3* and *CFHR1* and has been associated with AMD and aHUS [19], [20]. Because co-segregation of the *CFHR3-1*Δ deletion with the minor allele of rs6677604 in subjects with European Ancestry was observed in a previous study of AMD [20], we hypothesized that the association of *CFHR3-1*Δ with SLE was captured by SNPs in this study. Using multiplex ligation-dependent probe amplification (MLPA) (location of MLPA markers were shown in Figure 1A), we genotyped *CFHR3-1*Δ in 275 EA, 106 AA, 282 AS and 196 HS subjects, and then measured its LD with rs6677604. We found that *CFHR3-1*Δ and rs6677604 were in complete LD in EA (r^2^ = 1.00) and AS (r^2^ = 1.00), strong LD in HS (r^2^ = 0.85) and moderate LD in AA subjects (r^2^ = 0.60) (Table 2). In a subset of 58 unrelated AA subjects who were genotyped at both rs6677604 and rs16840639, we found that *CFHR3-1*Δ was in stronger LD with rs16840639 (r^2^ = 0.70) than with rs6677604 (r^2^ = 0.60). These results indicated that the association of the *CFHR3-1*Δ deletion with risk of SLE was tagged by the minor allele of rs6677604 in EA and rs16840639 in AA, respectively, suggesting that *CFHR3-1*Δ might be a risk variant for SLE.

**Table 2:** Pairwise LD between rs6677604 and *CFHR3-1*Δ in four ethnic groups.

	Genotype combination between rs6677604 and *CFHR3-1*Δ									Sample	Freq	
Ethnicity	11/ΔΔ	11/Δ+	11/++	12/ΔΔ	12/Δ+	12/++	22/ΔΔ	22/Δ+	22/++	size	of Δ	r^2^
EA	12	0	0	0	85	0	0	0	178	275	21.2%	1.00
AS	2	0	0	0	28	0	0	0	252	282	5.3%	1.00
HS	5	1	0	0	52	0	0	7	131	196	16.1%	0.85
AA	10	2	0	7	43	2	0	10	32	106	42.0%	0.60

Δ and + represent the presence and absence of *CFHR3-1*Δ, respectively. 1 and 2 represent the minor and major allele of rs6677604, respectively.

We showed that rs6677604 and *CFHR3-1*Δ were in the same block in AS (Figure 1B), and the minor allele of rs6677604 could perfectly tag the *CFHR3-1*Δ deletion (r^2^ = 1.00). Thus, the lack of significant association of rs6677604 with SLE in our previous AS dataset might be due to insufficient statistical power. To increase power, we further genotyped 787 Chinese SLE cases and 1065 Chinese controls and then assessed the association of rs6677604 with SLE in an enlarged AS dataset (2052 cases vs. 2325 controls). In the enlarged AS dataset, we detected the significant association of rs6677604 with SLE (MAF: 7.1% vs. 6.1%, *P* = 0.0485, OR[95%CI] = 1.19[1.00–1.40]), supporting the hypothesis that *CFHR3-1*Δ might also be a risk variant for SLE in the AS population.

### Tag SNPs suggested the *CFHR3-1*Δ deletion conferred a dosage-dependent risk effect of SLE

To test whether homozygous deletion of *CFHR3-1*Δ might confer a higher risk of SLE than heterozygous deletion, we compared the genotypic frequency of homozygous and heterozygous deletion to that of no deletion, respectively. In EA, using rs6677604 as a tag SNP, we found that the homozygous deletion of *CFHR3-1*Δ conferred a significantly increased risk of SLE (*P* = 7.5×10^−4^, OR[95%CI] = 1.47[1.17–1.84]) compared to no deletion, which was stronger than that of the heterozygous deletion (*P* = 0.0018, OR[95%CI] = 1.17[1.06–1.29]) (Table 3), suggesting a dosage dependent risk effect of the *CFHR3-1*Δ deletion. To confirm, we compared genotypic associations of *CFHR3-1*Δ in AS and AA using rs6677604 and rs16840639 as tag SNPs, respectively. In these two ethnic groups, we found that only homozygous deletion of *CFHR3-1*Δ conferred a significantly increased risk of SLE compared to no deletion (AS: *P* = 0.0021, OR[95%CI] = 3.30[1.47–7.41]; AA: *P* = 0.0011, OR[95%CI] = 1.40[1.14–1.71]) (Table 3), supporting the hypothesis that homozygous deletion of *CFHR3-1*Δ conferred a higher risk of SLE than heterozygous deletion. In a meta-analysis combining ORs of EA, AA and AS, we confirmed that the homozygous deletion of *CFHR3-1*Δ (*P*_meta_ = 3.2×10^−7^, OR[95%CI] = 1.47[1.27–1.71]) had a stronger association with risk of SLE than the heterozygous deletion (*P*_meta_ = 3.5×10^−4^, OR[95%CI] = 1.14[1.06–1.23]).

**Table 3:** Dosage-dependent risk effect of the *CFHR3-1*Δ deletion.

		Freq (number) of genotypes (11/12/22*)								
Ethnicity	Tag SNP	SLE			Control			Comparison	OR [95% CI]	*P*
EA	rs6677604	5.2%	35.5%	59.2%	3.8%	32.6%	63.5%	11 vs. 22	1.47 [1.17–1.84]	7.5×10^−4^
		(206)	(1398)	(2330)	(133)	(1137)	(2213)	12 vs. 22	1.17 [1.06–1.29]	0.0018
AS	rs6677604	1.1%	12.0%	86.8%	0.3%	11.5%	88.2%	11 vs. 22	3.30 [1.47–7.41]	0.0021
		(23)	(247)	(1782)	(8)	(267)	(2048)	12 vs. 22	1.06 [0.88–1.28]	0.51
AA	rs16840639	15.7%	43.6%	40.7%	12.5%	42.4%	45.2%	11 vs. 22	1.40 [1.14–1.71]	0.0011
		(261)	(725)	(676)	(237)	(806)	(859)	12 vs. 22	1.14 [0.99–1.32]	0.065
Combined								11 vs. 22	1.47 [1.27–1.71]	3.2×10^−7^
								12 vs. 22	1.14 [1.06–1.23]	3.5×10^−4^

*1 and 2 represent the minor and major allele of tag SNP, respectively.

### Tag SNPs suggested that *CFHR3-1*Δ was associated with SLE but not specific clinical manifestations preferentially

SLE is a complex disease with heterogeneous sub-phenotypes. To determine whether *CFHR3-1*Δ had a stronger association with specific clinical manifestations of SLE, we compared its frequency in SLE cases stratified by the presence or absence of each of the eleven ACR classification criteria (malar rash, discoid rash, photosensitivity, oral ulcers, arthritis, serositis, renal disorder, neurologic disorder, hematologic disorder, immunologic disorder and antinuclear antibody) and five autoantibodies (anti-dsDNA, anti-Sm, anti-RNP, anti-SSA/Ro and anti-SSB/La). In EA, we found that tag SNP rs6677604 of *CFHR3-1*Δ was associated with the absence of neurologic disorder (Table S2). However, in AA, we found that the corresponding tag SNP rs16840639 was associated with the absence of anti-dsDNA and the presence of serositis (Table S2), the latter of which was found not to be significant after Bonferroni correction for multiple comparisons. Insufficient clinical information for the majority of AS SLE patients precluded us from conducting these analyses. Taken together, these data did not provide evidence for a stronger association of *CFHR3-1*Δ with specific clinical manifestations of SLE.

## Discussion

In this study, we identified SLE-associated SNPs in the *CFH-CFHRs* region in three ethnic groups consisting of EA, AA and AS. In addition, we showed that the underlying causal variant was captured by rs6677104 and rs16840639 and could be localized to a ∼146 kb block extending from intron 9 of *CFH* to the 5′ region upstream of *CFHR4*. We demonstrated that the *CFHR3-1*Δ deletion, which has been associated with AMD and aHUS, could be tagged by the minor risk alleles of rs6677604 (r^2^ = 1.00 in EA and AS) and rs16840639 (r^2^ = 0.70 in AA) and showed dosage-dependent association with risk of SLE. These data strongly suggested that *CFHR3-1*Δ, which leads to reduced levels of CFHR3 and CFHR1 proteins, was the causal variant for increased risk of SLE within the SLE-associated block.

Multiple *CFH* exonic SNPs have been associated with various human diseases, but none of them were associated with SLE in this study. Y402H (rs1061170) is the most studied non-synonymous SNP of *CFH*. Y402H is located in SCR7 and affects the binding of CFH with glycosaminoglycans and CRP [22], [23], [24]. Y402H has been strongly associated with risk of AMD and MGPN2 but not associated with aHUS [16]. In this study, we genotyped a tag SNP of Y402H (rs7529589, r^2^ = 0.75 with Y402H according to HapMap CEU data) and detected no statistically significant association with SLE (Table S1). In a previous study, we had genotyped Y402H directly in 2033 EA cases and 2824 EA controls, and observed a similar result (37.4% vs. 37.7%, *P* = 0.81, OR = 0.99). I62V (rs800292) located in the N-terminal SCR2 is another well-studied non-synonymous SNP of *CFH*. Although I62V may result in increased binding of CFH with C3b and enhanced CFH co-factor activity and has been associated with decreased risk of AMD, MPGN II and aHUS [16], [25], it was not associated with SLE in this study (Table S1). D936E (rs1065489 in SCR16) was associated with lower host susceptibility to meningococcal disease in a recent GWAS [18]. We genotyped a perfect tag SNP (rs10489456) of D936E and failed to detect an association with SLE (Table S1). A synonymous SNP A474A (rs2274700 in SCR8) and its tag SNP rs1410996 were strongly associated with risk of AMD independent of Y402H [26], [27], but we detected only a marginal association between rs1410996 and risk of SLE in EA (Table 1), which was eliminated after conditioning on rs6677604 or rs16840639. In addition, two synonymous SNPs A307A (rs1061147 in SCR5) and Q672Q (rs3753396 in SCR13) that are in strong LD with Y402H and D936E, respectively, were not associated with SLE in our study. These data suggest that the previously described disease-associated *CFH* exonic SNPs do not contribute to the development of SLE.

Compared with SNP genotyping assays, genotyping assays for copy number variation are more labor-intensive and costly. Consequently, *CFHR3-1*Δ was not specifically genotyped in this study to assess its association with SLE. Instead, we evaluated the effect of the *CFHR3-1*Δ deletion on SLE development indirectly using tag SNPs that were in strong LD with it. We first confirmed that *CFHR3-1*Δ was in strong LD with rs6677604 in EA, similar to previous studies of AMD [20], [28]. Furthermore, we showed that *CFHR3-1*Δ was also in strong LD with rs6677604 in AS and HS. In addition, we found that *CFHR3-1*Δ was in stronger LD with rs16840639 than with rs6677604 in AA. Of note, in AA, the most significant association with SLE was detected at rs16840639 rather than rs6677604, and the risk haplotype H1 in AA was perfectly tagged by the minor allele of rs16840639 rather than rs6677604 (Figure 2), suggesting that rs16840639 captured the underlying causal variant *CFHR3-1*Δ in AA. Using these tag SNPs, we deduced that homozygous *CFHR3-1*Δ deletion conferred higher risk of SLE than heterozygous deletion, which suggested a change in gene dosage of the encoded proteins CFHR3 and CFHR1 might account for the increased SLE risk.

The *CFHR3-1*Δ deletion was associated with the general phenotype of SLE but did not consistently exhibit stronger signals to a specific clinical manifestation in EA and AA, and was not specifically associated with the presence of renal disorder. This is in contrast to the effect of CFH deficiency, which results in the development of glomerulonephritis in *CFH* knockout mice due to uncontrolled C3 activation [11], [29]. In addition, the absence of CFH in plasma causes human MPGN II [30], but an association of the *CFHR3-1*Δ deletion with MPGN II has not been reported. The absence of an association of *CFHR3-1*Δ with renal disorder in lupus suggests that *CFHR3* and *CFHR1* play a different role from *CFH* in the pathogenesis of lupus, although further studies are required to validate the lack of association between the *CFHR3-1*Δ deletion and renal disorder in SLE.

The *CFHR3-1*Δ deletion has opposite effects in different diseases [9], and the underlying mechanism is poorly understood. Activated complement pathways converge to generate C5 convertase, which cleaves C5 into C5a and C5b. C5a is a potent chemoattractant. C5b initiates the formation of the terminal MAC. CFHR1 acts as a complement regulator to inhibit C5 convertase activity and terminal MAC formation [10], and CFHR3 displays anti-inflammatory effects by blocking C5a generation and C5a-mediated chemoattraction of neutrophils [31]. Increased neutrophils lead to inflammatory injuries in many non-infectious human diseases [32]. It has been shown that immune complex-induced inflammatory injuries are largely mediated by C5a receptor and blocking C5a receptor reduces manifestation of lupus nephritis in mice [33], [34]. In addition, increased apoptotic neutrophils contribute to autoantigen excess and have been associated with increased disease activity in SLE [35]. The *CFHR3-1*Δ deletion results in decreased CFHR3 and CFHR1 levels and may therefore lead to uncontrolled production of chemoattractant C5a predisposing to SLE. Of interest, the *CFHR3-1*Δ deletion also has a risk effect in aHUS and the CFHR3 and CFHR1 deficiency in plasma has been associated with the presence of anti-CFH autoantibodies, which bind to the C-terminus of CFH and block CFH binding to cell surfaces [36], [37]. It is also possible that *CFHR3-1*Δ is also associated with the presence of anti-CFH autoantibodies in SLE and thus leads to impaired CFH cell surface regulation.

Both CFHR3 and CFHR1, lacking the CFH N-terminus regulatory activity, were reported to compete with CFH for binding to C3b, and thus CFHR3 and CFHR1 deficiency may lead to enhanced CFH regulation [31], which may explain the protective effect of the *CFHR3-1*Δ deletion in AMD. Of interest, as mentioned before, the non-synonymous SNP I62V in the CFH regulatory domain may also increase CFH regulation. I62V confers a protective effect in AMD, aHUS and MPGN II [16], but it was not associated with SLE in this study.

Statistical under-powering might account for the failure to detect a significant association in HS dataset. First, rs6677604 and *CFHR3-1*Δ were in strong LD and could be defined into a block in HS, which excluded the possibility that the *CFHR3-1*Δ deletion was not tagged in the HS dataset. In addition, there was no genetic heterogeneity of rs6677604 in the four ethnic groups (*P* = 0.76), in which the risk minor allele showed consistently higher frequency in cases than in controls. Finally, based on rs6677604, post hoc analysis indicated a much lower power of 51% in HS to detect association with SLE (*P*<0.05) than the power of 98% in EA and 92% in AA. Thus, the association of *CFHR3-1*Δ with SLE in HS needs to be further evaluated in a larger dataset.

One limitation of this study is that we have not addressed whether rare variants in the *CFH*-*CFHRs* region may contribute to the development of SLE. Pathogenic rare variants clustering in CFH C-terminus affect CFH cell surface binding, but they were only found in aHUS patients, not in AMD, MPGN II patients and healthy controls [16]. Deep sequencing of exons in CFH C-terminus in patients with SLE may elucidate whether these rare variants are associated with SLE.

To our knowledge, this study is the first to show that genetic variants in the *CFH-CFHRs* region are associated with SLE susceptibility. Our consistent observations of dose-dependent association between *CFHR3-1*Δ and SLE across three distinct ancestral populations and no association in *CFH* exonic SNPs suggest a novel role for CFHR3 and CFHR1 in the pathogenesis of SLE. Further functional studies are required to elucidate the underlying mechanism of *CFHR3-1*Δ.

## Materials and Methods

### Ethics statement

The study was approved by the Human Subject Institutional Review Boards or the ethnic committees of each institution. All subjects were enrolled after informed consent had been obtained.

### Subject collection

To test the association of *CFH* and *CFHRs* with SLE, we used a large collection of samples from case-control subjects from multiple ethnic groups. These samples were from the collaborative Large Lupus Association Study 2 (LLAS2) and were contributed by participating institutions in the United States, Asia and Europe. According to genetic ancestry, subjects were grouped into four ethnic groups including European American (3,936 cases vs. 3,491 controls), African American (1,679 cases vs. 1,934 controls), Asian (1,265 cases vs. 1,260 controls) and Hispanic enriched for the Amerindian-European admixture (1,492 cases vs. 807 controls). Asians were comprised of Koreans (884 cases vs. 994 controls), Chinese (200 cases vs. 205 controls) and subjects from other East Asian countries such as Japan and Singapore (181 cases vs. 61 controls). African Americans included 275 Gullahs (152 cases vs. 123 controls), who are subjects with African Ancestry.

To test LD between *CFHR3*-1Δ and SLE-associated SNPs, we used 275 unrelated European Americans (187 cases vs. 88 controls), 106 African Americans (88 unrelated subjects [58 cases vs. 30 controls] and 18 subjects from 6 SLE trios families), 282 unrelated Chinese (218 cases vs. 64 controls) and 196 Hispanics (157 unrelated subjects [91 cases vs. 66 controls] and 39 subjects from 13 SLE trios families). All of these subjects were enrolled from UCLA.

To enlarge the sample size of Asians for association test, we used 1,852 Chinese case-control subjects (787 vs. 1065) recruited from Shanghai Renji Hospital, Shanghai Jiao Tong University School of Medicine.

All SLE patients met the American College of Rheumatology (ACR) criteria for the classification of SLE [38].

### SNP genotyping and data cleaning

LLAS2 samples were processed at the Lupus Genetics Studies Unit of the Oklahoma Medical Research Foundation (OMRF). SNP genotyping was carried out on the Illumina iSelect platform. Subjects with individual genotyping call rate <0.90 were removed because of low data quality. Subjects that were duplicated or first degree related were also removed. Both principal component analysis and global ancestry estimation based on 347 ancestry informative markers were used to detect population stratification and admixture, as described in another LLAS2 report [39]. After removing genetic outliers, a final dataset of 15,864 unrelated subjects (8,372 cases vs. 7,492 controls) was obtained.

Taqman SNP genotyping assay (Applied Biosystems, California, USA) was used to genotype rs6677604 for subjects who were not recruited into LLAS2.

### MLPA genotyping

MLPA kit “SALSA MLPA KIT P236-A1 ARMD mix-1” was used to genotype the *CFH-CFHRs* region according to the manufacture's instruction (MRC-Holland, Amsterdam, The Netherlands). ABI 3730 Genetic Analyzer (Applied Biosystems) was used to run gel electrophoresis. Software Peak Scanner v1.0 (Applied Biosystems) was used to extract peaks generated in electrophoresis. Coffalyser v9.4 (MRC-Holland) was used to readout copy number of target region.

### Statistical analysis

The HWE test threshold was set at *P*>0.01 for controls and *P*>0.0001 for cases. SNPs failing the HWE test were excluded from association test. SNPs showing genotyping missing rate>5% or showing significantly different genotyping missing rate between cases and controls (missing rate>2% and *P*_missing_<0.05) were also excluded from association test. In allelic association test (Pearson's χ2–test), the significance level was set at *P*<0.05. Haploview 4.2 was used to estimate pairwise LD values between SNPs, define haplotypes blocks and calculate haplotypic association with SLE. Haplotype-based conditional association analysis was carried out by Plink v1.07. Mantel-Haenszel analysis was performed to generate the meta-analysis *P* value. CaTS was used to calculate statistical power.

## Supporting Information

Table S1 Allelic association between 60 tested SNPs and SLE in all four ethnic groups.
(XLS)

Table S2 Association of *CFHR3-1Δ* with clinical manifestations of SLE.
(XLS)
